# Can Daytime Napping Assist the Process of Skills Acquisition After Stroke?

**DOI:** 10.3389/fneur.2018.01002

**Published:** 2018-11-22

**Authors:** Winifried Backhaus, Hanna Braass, Christian Gerloff, Friedhelm C. Hummel

**Affiliations:** ^1^Department of Neurology, University Medical Center Hamburg-Eppendorf, Hamburg, Germany; ^2^Defitech Chair of Clinical Neuroengineering, Brain Mind Institute and Center for Neuroprosthetics, Swiss Federal Institute of Technology (EPFL), Geneva, Switzerland; ^3^Defitech Chair of Clinical Neuroengineering, Brain Mind Institute and Center for Neuroprosthetics, Swiss Federal Institute of Technology Valais (EPFL Valais), Clinique Romande de Réadaptation, Sion, Switzerland; ^4^Clinical Neuroscience, Medical School University of Geneva, Geneva, Switzerland

**Keywords:** stroke, motor recovery, plasticity and learning, napping, aging, consolidation, motor adaptation

## Abstract

Acquisition and reacquisition of skills is a main pillar of functional recovery after stroke. Nighttime sleep has a positive influence on motor learning in healthy individuals, whereas the effect of daytime sleep on neuro-rehabilitative training and relearning of the trained skills is often neglected. The aim of this study was to investigate the relationship between daytime sleep (napping) and the ability to learn a new visuomotor task in chronic stroke patients. The main hypothesis was that sleep enhances motor memory consolidation after training resulting in better motor performance after a period of daytime sleep. Thirty stroke survivors completed the study. They were randomized to one of three different conditions (i) wakeful resting, (ii) short nap (10–20 min), or (iii) long nap (50–80 min). All individuals trained the task with the contralesional, stroke-impaired hand, behavioral evaluation was performed after the break time (wake, nap), and 24 h later. Patients demonstrated a significant task-related behavioral improvement throughout the training. In contrast to the main hypothesis, there was no evidence for sleep-dependent motor consolidation early after the initial, diurnal break, or after an additional full night of sleep. In a secondary analysis, the performance changes of stroke survivors were compared with those of a group of healthy older adults who performed the identical task within the same experimental setup with their non-dominant hand. Performance levels were comparable between both cohorts at all time points. Stroke-related difficulties in motor control did not impact on the degree of performance improvement through training and daytime sleep did not impact on the behavioral gains in the two groups. In summary, the current study indicates that one-time daytime sleep after motor training does not influence behavioral gains.

## Introduction

Stroke is one of the main causes of acquired disability in adulthood (1). Individuals and their caregivers are confronted with deficits affecting multiple domains of daily life. Latter includes loss in movement control, which is where common physical rehabilitation programs come to place.

Individual rehabilitation success is to a relevant degree dependent on re-learning of motor skills. Motor learning is used as an umbrella term which includes the learning of a new motor skill during and after task performance and the adaptation of motor skills (2). Motor consolidation is a process during which this new information is consolidated into existing long term storage. Latter, with the goal of making it resistant to interference (3). The magnitude of motor learning depends on several factors, one of them may be sleep (4, 5). Some sleep-studies point to a performance-enhancing effect in younger and older adults (6–15) while others do not (12, 16–22). Also, well-recovered individuals after stroke, who practice a sequence task show performance-enhancing effects of a night of sleep (21, 22). A number of studies underlining this finding (21, 23–25) report performance improvements after a night of sleep and no improvements after an equivalent period of wakefulness during the day. Not only long sleep durations, such as overnight sleep but also shorter sleep periods, such as daytime naps are subject of research. Support that naps might facilitate the effects of motor learning in healthy young adults has been well documented (26–31). A similar positive effect was reported in older adults after an additional night of sleep (32). Comparable studies in individuals after stroke are lacking, but less disturbed sleep seems to be associated with better recovery (33).

The term motor adaptation implies that an automatized performance has to be adapted to alterations in the intrinsic or extrinsic coordinate system (2). With the adjustment of movements to new demands being part of daily living, performance improvements of individuals after stroke are assumed to be present during training in all groups, after sleep or wakefulness. Based on the results of previous publications (21, 24, 25) finding performance improvements over-night but not during wakefulness, longer nap durations are expected to lead to greater performance changes than shorter nap durations, while shorter nap durations are expected to be superior to equivalent periods of wakefulness. The central hypothesis of this study was that daytime naps after training could accelerate the learning rate and therefore enhance motor performance in stroke survivors. If so, this could be used as an adjuvant tool in motor rehabilitation after stroke. Within the present daytime nap approach performance of a visuomotor task is compared after (i) a period of wakefulness with performance after (ii) a short (10–20 min) nap or (iii) after a longer (50–80 min) nap period. The task integrated visual feedback, visual motor perturbation and equivalent motor planning (34), and was performed with the affected hand, i.e., the hand which is controlled by the lesioned hemisphere. In a secondary analysis, the observed performance changes in stroke survivors were compared with those of a previously studied group of healthy adults (35).

The overarching aim of the present study is to provide a more in-depth understanding of performance and consolidation in a visuomotor adaptation task in individuals after stroke and the influence of daytime napping.

## Methods

### Participants

Thirty patients at least 6 months after a first-ever, mono-hemispheric ischemic or hemorrhagic stroke participated in the study. All participants performed the task with the contralesional, stroke-impaired hand and gave written informed consent prior to participation. Neuropsychological or physiological constraints limiting correct task execution were reasons for exclusion. The latter include symptoms such as hemianopia, apraxia, aphasia, neglect, pain, or spasticity. A number of tests assisted screening for depressive symptoms [Becks Depression Inventory, BDI (36)], state of cognition [Mini Mental State Examination, MMSE (37)], upper limb function [gross motor function: Fugl-Meyer Upper Extremity score (F-M 38)], fine motor function: the nine-hole peg-test [9HPT (39)] and sleep quality [Pittsburg Sleep Quality Index, PSQI (40)] There was no prior polysomnographic recording to exclude sleep related disorders. Date of stroke and stroke-affected brain regions were extracted from medical records or determined from magnetic resonance imaging. The comparison group of 30 healthy older adults, previously described elsewhere (35) participated in an identical experimental setup. The study was approved by the local ethics committee (“Ethik-Kommission der Ärztekammer Hamburg,” Germany, PV4596) in accordance with the declaration of Helsinki (41).

### Groups

**Figure 1:**
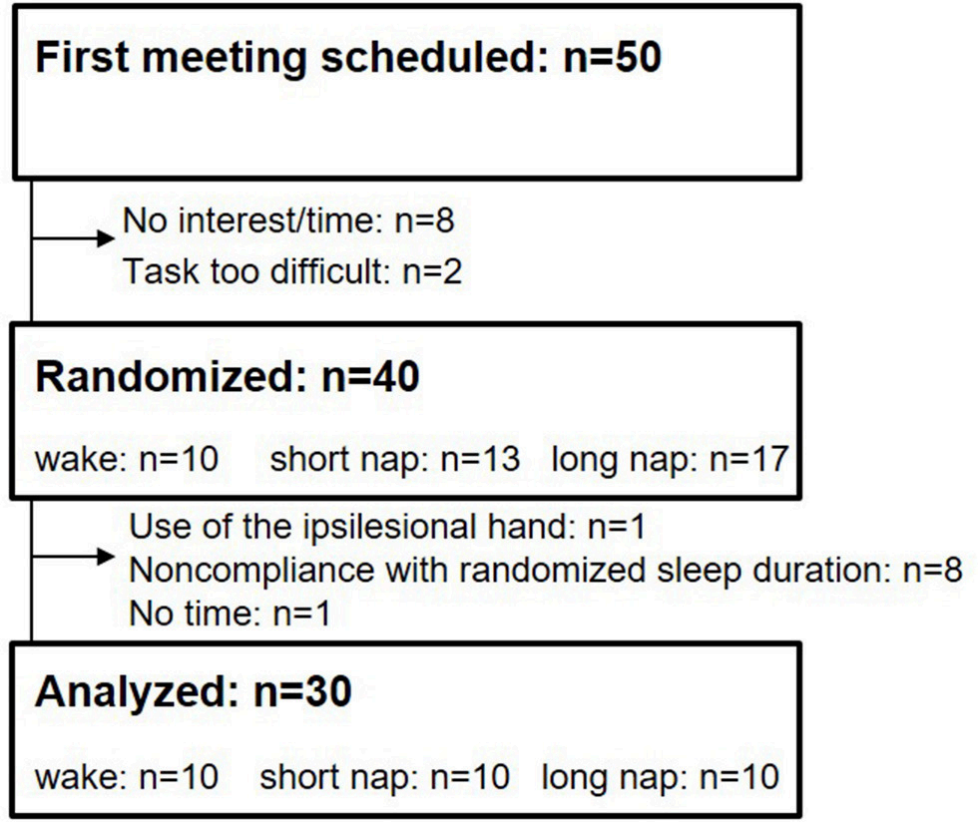
Flowchart of participant recruitment.

An initial meeting to help participants get acquainted to the sleep laboratory, the study setup and the task, was scheduled with 50 prospective participants (Figure 1). Two participants were not capable to perform the task and eight others declined due to time constraint. All remaining 40 participants were randomized to one of three groups, (i) wake, (ii) short nap, and (iii) long nap, by applying the sealed-envelope method. In case of non-compliance with the assigned sleep group (participants were not able to sleep the required amount of time), the group-assigning envelope was returned to the remaining entity and the participant was excluded from the analysis (*n* = 8). Participants randomized to the wake group stayed awake for 45-min between the first two learning sessions. During this time they were seated upright in a comfortable chair to watch historical movies while polysomnography was performed to rule out sleep. Participants in the short nap group were given a 45-min and participants in the long nap group a 90-min nap opportunity between the first and second learning session. Only participants napping between 10 and 20 min in the short nap group, and 50–80 min in the long nap group, and remaining awake in the wake group were included for all further analysis (Figure 1). Additional 30-min of wakeful rest (upright) were given to participants in the long nap group, to recover from possible effects of sleep inertia. All participants were instructed to refrain from naps outside the limits of the study protocol and from alcohol or caffeine on the day the learning sessions took place.

### Study protocol and design

**Figure 2:**
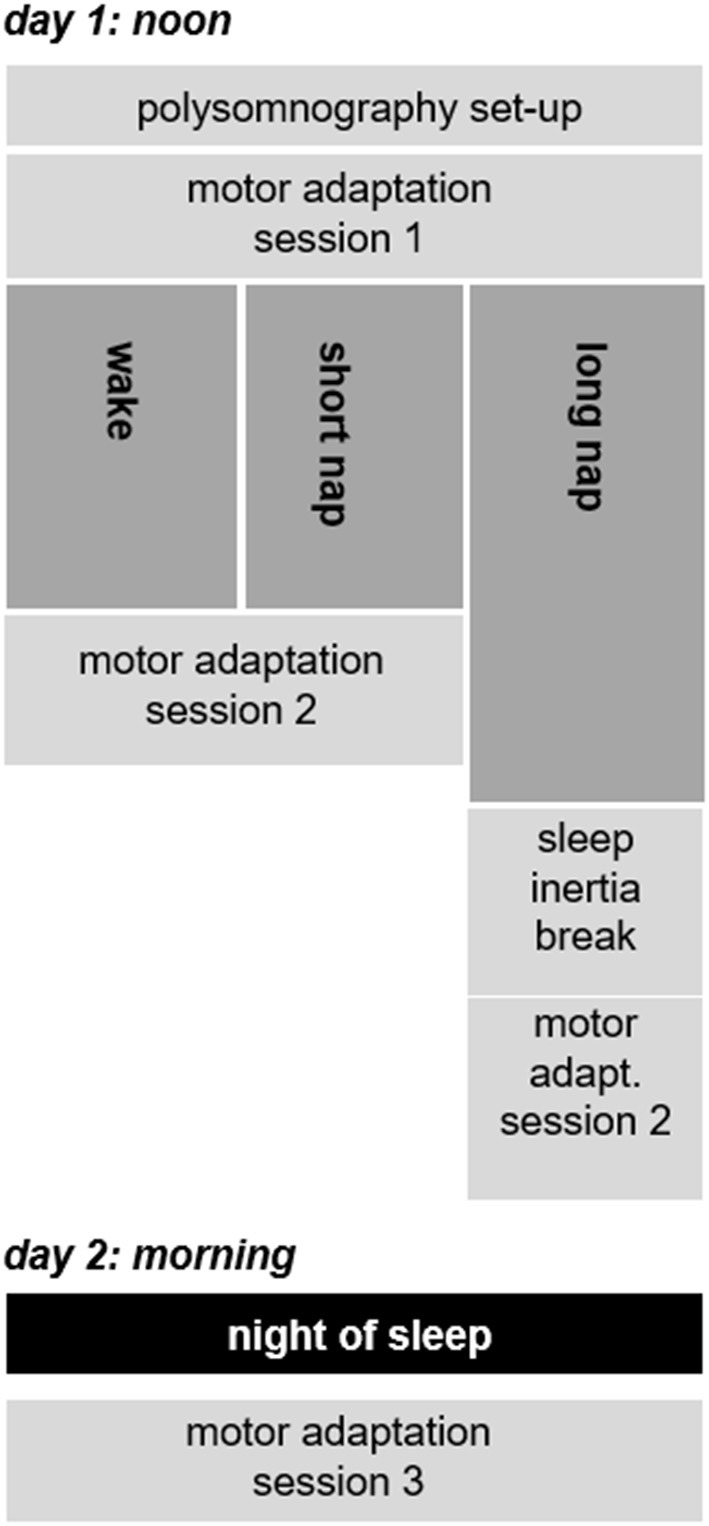
Timeline of the study design.

The study design and the adaptation task was identical to previously published studies in younger and older adults (35, 42). In brief, this included three successive learning sessions taking place at the University Sleep Medical Center Hamburg (Figure 2). The first session around noon was followed by a second session after a midday break. A final session, after a night of sleep, concluded the experiment. The time between the first two sessions on day 1 was recorded with polysomnographic measures (Alice 3.5, Respironics Inc.), irrespective of group allocation. The polysomnographic data were subsequently analyzed according to the guidelines of the AASM (43), once on a single-subject level on the day of recording and once more after all participants completed the study. While the first staging ensured homogenous sleep durations across groups (NREM 2 as indication of sleep start) only the results from the last staging were incorporated in all further analyses. This last staging, performed after all 30 participants had completed the study, reduced possible day-to-day scoring variability. The level of sleepiness was rated subjectively [Stanford Sleepiness Scale, SSS (44)] prior to each adaptation learning session and quantified with a reaction time test [as also done in (42)]. Latter measured the time participants required to react to a visual stimulus with a button press. Similar to previous publications, the visual stimuli appeared at random intervals (6–8 s). The time each individual required (between the visual stimulus and the button press) was used to quantify the level of vigilance (45, 46).

### Motor learning task

**Figure 3:**
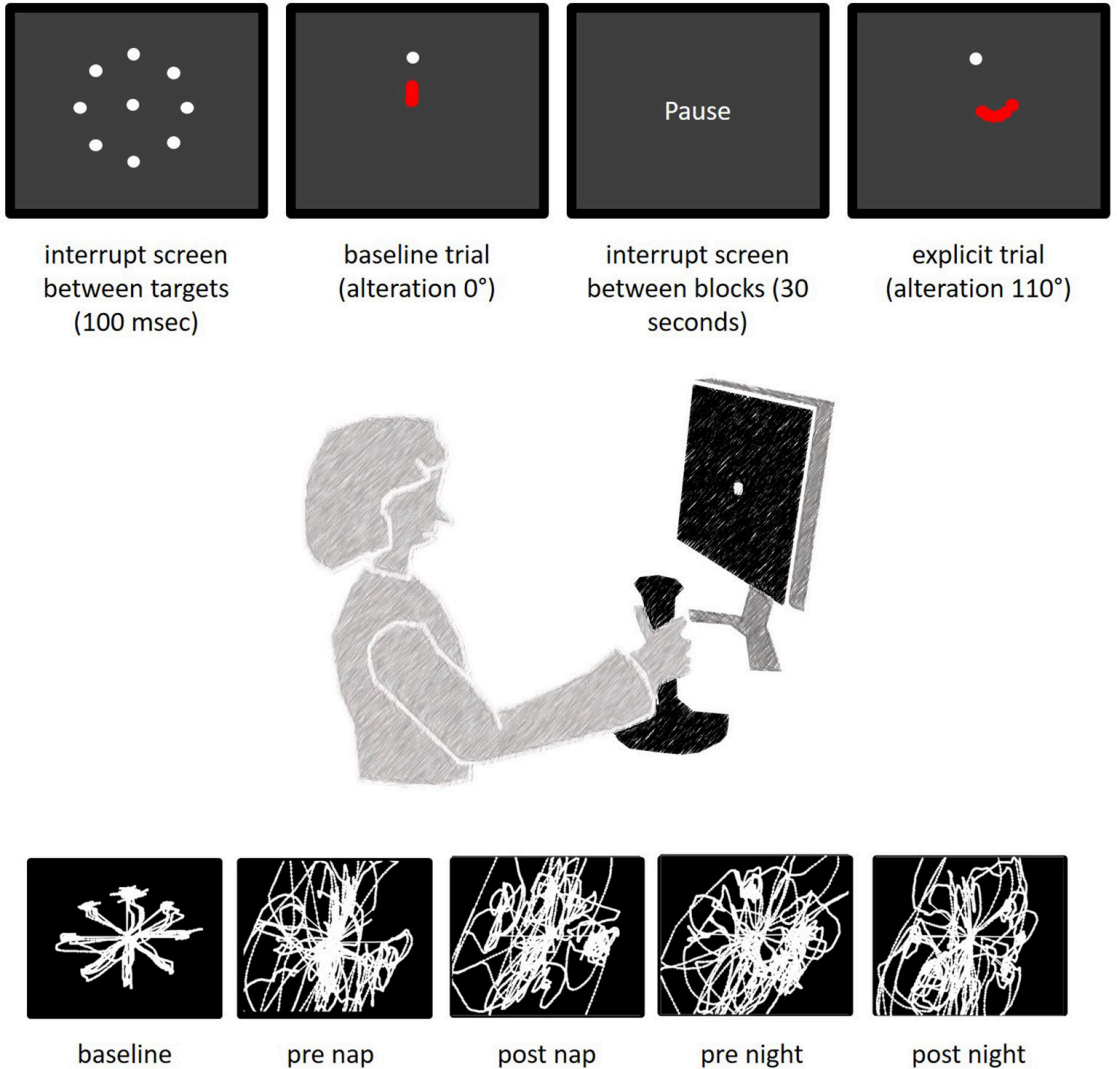
Task description. The top images present the screen display between trials (interrupt screen), during baseline and during the explicit trials. The red dots symbolize the movements of the dot-cursor. Latter was actually white and originated from the middle of the screen (see interrupt screen). The bottom images represent typical cursor paths of individuals after stroke at different times of the experiment.

The participants sat in front of a 70 Hz screen and were instructed to collect targets (30 pixel sized dots) by moving a joystick with the contralesional (stroke impaired) hand (35, 42) [similar to (27)]. Joystick movements were in turn projected on a the screen as a dot-cursor (Figure 3). The target disappeared after the dot-curser remained within a radius of 12 pixels from its center for at least 100 ms. As soon as the cursor reached its neutral position in the middle of the screen a new target appeared at one of eight predefined target locations. Three baseline blocks, each block lasting for 150 s with an additional 30 s break, and a preceding training helped participants adjust to the joystick handling. The joystick movement trajectory was altered by 110° after completing the baseline trails. Moving the joystick to the 12-o'clock position induced cursor movements rotated by 110°, which was visible on the screen as a movement to the bottom right. Each session included one random block during which the joystick alteration was changed to 300°, 60°, or 290° (sessions 1–3). The outcome measure was the number of collected targets.

### Statistical analyses

Data from participants unable to sleep as randomized were excluded prior to the analyses. A repeated-measures mixed model approach with pre-defined *post-hoc* testing was performed in SPSS (SPSS Statistics 23) and R (3.3.2). Each session included 6 blocks of learning of which the first and the last block were analyzed. Learning changes over all three sessions included a total of 6 individual learning blocks. Sleep duration was defined as fixed effect and participant as the random effect. The initial model contained all additive main effects for dependent and the interaction terms for independent variables. To exclude possible effects related to the initial motor capacity of individuals during task execution, the baseline performance, prior to the actual learning task, was included as a covariate. Baseline measures were compared using simple *t*-tests adjusting alpha with Bonferroni-correction for multiple comparisons. Offline gain was defined as the difference in means between the last and the first learning block encircling either the diurnal or the nighttime break. Analyses combining data from stroke and older adults were carried out by nesting the groups within their respective population. Based on means and standard deviations of a study on sleep-induced improvement of implicit motor learning in individuals after stroke (21), with a group size of *n* = 10 per trial arm, the statistical power to detect a relevant effect was 70%. Significance level in the current study was set to α = 0.05. All data are expressed as mean ± standard deviation unless otherwise indicated.

## Results

### Baseline data

Of 50 contacted stroke survivors, 30 participants, between the ages of 46 and 82 (62.0 ± 7.7 years), completed the motor adaptation task as randomized (Figure 1). The randomized groups were similar at baseline (*p* > 0.124) concerning the following criteria: age, sleep quality (PSQI), depression (BDI), handedness (Oldfield), cognition (MMSE), and baseline motor performance. Lesion sides were equally distributed with 16 left– and 14 right–hemispheric stroke patients (Table 1, Supplementary Table 1). Learning took place on 2 consecutive days (Figure 2); the first learning session started around 1 p.m. (between-group difference: *p* = 0.077) and the final learning session started around 10:20 a.m. (between-group difference: *p* = 0.827) the following day.

**Table 1:** Baseline and stroke characteristics.

	**Awake**	**Short nap**	**Long nap**	***p***
*N*	10	10	10
Age (years)	59.7 ± 5.5	60.0 ±12.1	66.3 ± 5.5	0.167
Female	5	3	1	0.159^a^
Time since stroke (months)	45.6 ± 33.5	36.6 ± 28.0	65.26 ± 70.3	0.404
Affected hemisphere = left	6	4	6	0.596^a^
Stroke in the dominant hemisphere	6	3	7	0.186^a^
F-M score (upper extremity)	50.2 ±18.0	57.5 ±12.0	57.1 ± 7.9	0.399
9HPT (s)	30.3 ± 6.5	37.1 ± 33.3	53.6 ± 76.0	0.662
Unable to perform the 9HPT (*n*)	4	1	2	0.283^a^
number of targets at baseline	35.2 ± 18.5	46.8 ± 12.3	43.2 ± 13.8	0.233

*Lesion location and functional classification of participants*.

a *p-values are based non-parametric testing (Kruskal-Wallis)*.

### Sleep-related variables

Participants in the awake group were asked to maintain their regular sleep schedule whereas participants in the short nap and the long nap group were asked to shorten the nighttime sleep duration prior to the nap the following day. This could be achieved either by going to bed later on the day prior to or by rising earlier on the day of testing. In the night prior to the experiment participants slept around 8 h (awake 565.0 ± 297.6 min, short nap 459.0 ± 103.2 min, long nap 418.5 ± 99.4 min). The levels of sleepiness scored on a 7-point Likert scale (SSS), was comparable between groups [Kruskal-Wallis H test $\chi_{\left(2\right)}^{2}$ = 0.7, *p* = 0.702]. Participants in the wake group stayed awake for 46.4 ± 2.9 min until starting the following learning session. Participants in the short nap group slept for 15.5 ± 3.0 min, and participants in the long nap group for 63.2 ± 8.2 min (Table 2). After this period of rest, significant differences in levels of sleepiness (SSS) emerged [χ${}_{\left(2\right)}^{2}$ = 9.4, *p* = 0.009]. Participants that had remained awake felt sleepier than those participants given the opportunity to sleep. This subjective level of sleepiness was not included as cofactor into further analyses, as it failed to enhance any of the below calculations significantly. In addition, this difference vanished after a night of sleep [prior session 3: χ${}_{\left(2\right)}^{2}$ = 2.9, *p* = 0.238]. To quantify the level of sleepiness, a visual reaction time test was performed prior to each session. A repeated-measures ANOVA with Huynh-Field correction did not show significant effects of time *F*_(1.8, 49)_ = 2.6, *p* = 0.090 and no significant interaction of group and time [*F*_(3.6, 49)_ = 1.5, *p* = 0.218] for reaction times prior to session 2.

**Table 2:** Sleep duration of individuals after stroke in minutes (means and standard deviations).

	**Wake**	**Stage N1**	**Stage N2**	**Stage N3**	**REM**	**TST (nap)**	**TST (night)**
Awake	46.4 ± 2.9	0.0 ± 0.0	0.0 ± 0.0	0.0 ± 0.0	0.0 ± 0.0	0.0 ± 0.0	423.5 ± 75.0
Short nap	36.5 ± 12.9	6.4 ± 4.5	7.6 ± 4.1	1.6 ± 4.1	0.0 ± 0.0	15.5 ± 3.0	465.0 ± 82.8
Long nap	55.5 ± 9.7	20.5 ± 13.3	32.0 ± 11.9	10.8 ± 15.2	0.0 ± 0.0	63.2 ± 8.2	447.5 ± 83.8

*The total sleep time (TST) is derived from polysomnographic measures (nap) and from retrospective sleep logs (night)*.

### Performance changes within the stroke population

All groups started from a comparable baseline level (*p* = 0.233), collecting around 42 targets within a 150 s movement period. The participants increased their motor performance over time [main effect of learning *F*_(5, 135)_ = 25.3, *p* < 0.001]. The significant main effect of the baseline score [*F*_(1, 26)_ = 19.1, *p* < 0.001] on this motor performance change, was included as a covariate in all further analyses. The F-M score was not included as a covariate as it did not enhance the model and was significantly correlated with the baseline performance (Pearson *p* ≤ 0.001, *R*^2^ = 0.50).

**Figure 4:**
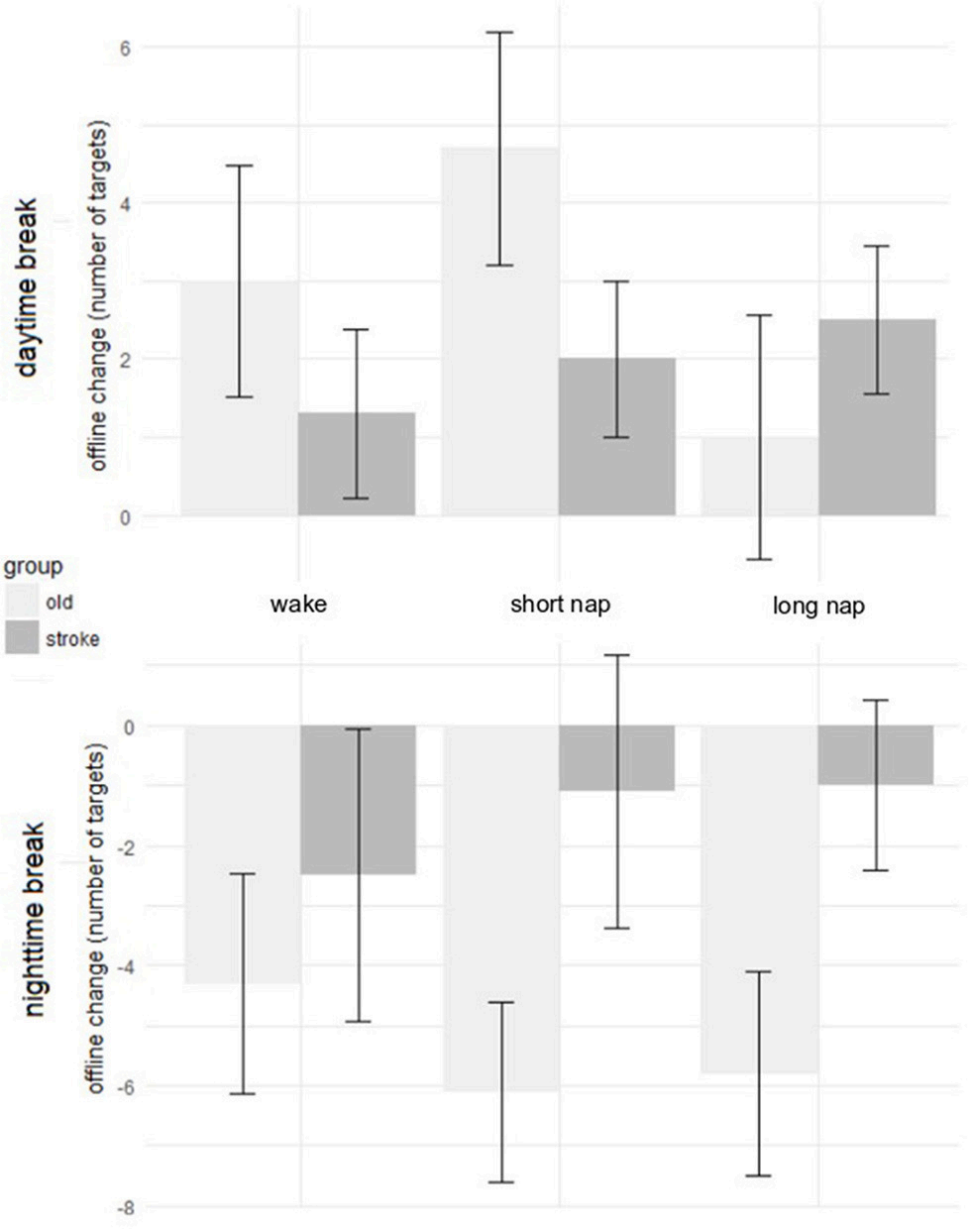
Offline change during daytime rest **(Top)** and after a night of sleep **(Bottom)** in healthy older adults (light gray) and individuals after stroke (dark gray). The error bars represent the standard error of the mean.

There was no significant difference in learning over the three sessions between the groups [group^*^time *F*_(10, 135)_ = 0.3, *p* = 0.983] and also not within the three sessions (online learning) [group^*^time *F*_(4, 54)_ = 0.5, *p* = 0.749]. To determine the performance changes between the sessions (offline learning) a second calculation included only the trials directly encircling these intervals. Performance improvements dominated the daytime break (awake: +1.3 ± 3.4, short nap: +2.0 ± 3.2, long nap: +2.5 ± 3.0 targets) and performance deteriorations were found after the longer nighttime break (awake:−2.5 ± 7.7, short nap:−1.1 ± 7.2, long nap:−1.0 ± 4.5 targets). The numeric trend toward larger improvements during the day and less deterioration after a night of sleep in the daytime sleep groups did not reach significance [group^*^time *F*_(2, 87)_ = 1.7, *p* = 0.185]. Daytime and nighttime offline learning differed significantly, as seen in the significant main effect for the change over time [*F*_(1, 27)_ = 6.7, *p* = 0.015]. *Post-hoc*, this could not be specified on group level–there was no significant difference between the groups for offline change when comparing day and night (*p* = 0.113) (Figure 4).

### Performance changes of older adults and stroke patients

In a second step, the dataset was supplemented by results from a population of healthy individuals (71.5 ± 5.6 years), who similarly showed no differences in performance when comparing the groups wake, short nap and long nap (35). Both populations (older adults and stroke patients) achieved significant learning improvements over time [main effect: *F*_(5, 290)_ = 92.1, *p* < 0.001]. The latter were comparable between populations. A significant main effect for population was absent [*F*_(1, 57)_ = 0.1, *p* = 0.777]. *Post-hoc* testing of the interaction between the population and learning over time [*F*_(5, 290)_ = 3.2, *p* = 0.007] revealed no significant differences between both populations at any time point (*p* > 0.103). The significant interaction was solely driven by the magnitude of performance change over time–performance increase and decline which differed per population. The baseline score was a significant cofactor for learning [main effect: *F*_(1, 57)_ = 38.8, *p* < 0.001].

### Offline changes of older adults and stroke patients

**Figure 5:**
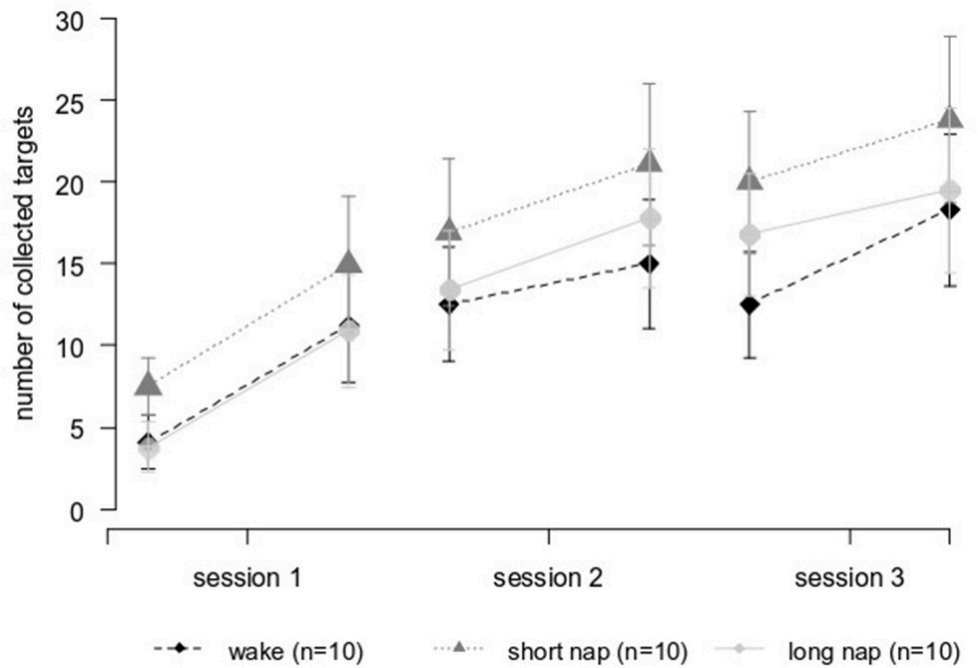
Performance improvements over time in individuals after stroke. Session 1 and 2 were interleaved by the midday break (wake and nap). A night of sleep separated session 2 and session 3.

The offline changes during day- and nighttime were calculated by subtracting the raw values of the learning blocks encircling the breaks (Table 3). There was no significant main effect for group (wake, short nap, long nap) nested within the population (stroke, old) [*F*_(2, 53)_ = 0.9, *p* = 0.408], nor for the baseline score [*F*_(1, 53)_ = 0.01, *p* = 0.912]. The factor time, differentiating between daytime and nighttime offline learning, was significant as a main effect [*F*_(1, 56)_ = 40.4, *p* > 0.001] and also in the interaction with the population [*F*_(1, 56)_ = 6.8, *p* = 0.012], but not in interaction with the group [*F*_(2, 56)_ = 0.347, *p* = 0.708]. From the latter, it can be concluded that, also in larger samples, there is no significant effect of midday sleep on change of motor performance. All groups, within one population, showed similar behavioral changes. The *post-hoc* analyses of the interaction term time^*^population underlined that performance change differed significantly between the daytime and nighttime change, in both populations (*p* < 0.012). This was expected as there was a general trend toward performance improvements during daytime and performance stagnation and reduction during the nighttime break. There is no difference in daytime change between older adults and individuals after stroke (*p* = 0.463). However, the overnight performance change differed significantly for individuals after stroke and healthy older adults (*p* = 0.005). This difference emerged as older adults deteriorated significantly after a night of sleep, whereas individuals after stroke were able to maintain their performance level throughout a day- and nighttime break (Figure 5).

**Table 3:** Healthy older adults and individuals after stroke: comparison of performance scores over time, within populations (merged groups) and between.

	**Baseline**	**Pre-break 1**	**Post-break 1**	**Daytime change**	**Pre-break 2**	**Post-break 2**	**Nighttime change**
Stroke	41.7 ± 15.4	12.3 ± 11.6	14.3 ± 12.1	*p* = 0.414	18.0 ± 13.7	16.4 ± 12.0	*p* = 0.891
Old	47.8 ± 10.7	13.2 ± 9.1	16.1 ± 9.9	*p* = 0.040	23.7 ± 10.0	18.3 ± 10.2	*p* < 0.001
Stroke vs. Old	–	–	–	*p* = 0.463	–	–	*p* = 0.005

*Higher scores are equivalent to better performance*.

## Discussion

This study compared the role of daytime napping in a visuomotor adaptation task in chronic stroke survivors and healthy older adults. The aim was to investigate whether daytime napping can assist visuomotor adaptation learning when adapting to an externally induced visual rotation. Both, older adults and individuals after stroke, were able to adapt their movements and thereby improve their performance significantly over time, irrespective of daytime sleep or daytime wakefulness. A clear distinction between both populations concerned the magnitude of offline performance change over the following night of sleep. Performance deteriorated significantly in healthy older adults, irrespective of daytime sleep or daytime wakefulness, a result not present in individuals after stroke.

Based on prior studies evaluating the effect of sleep on motor learning in individuals after stroke (21–23, 25), it was anticipated that performance levels after sleep would be superior to those reached after wakeful rest. This hypothesis could not be confirmed. A range of factors related to sleep (sleep duration, sleep stages, sleep depth) or to the sample population may have influenced the results and will be highlighted in the following. In addition, the questions why offline changes differed between daytime and nighttime break and why older adults deteriorated more over a night of sleep than individuals after stroke will be discussed.

### Sleep-related aspects

This study incorporated two periods of rest, daytime, and nighttime. Positive sleep-dependent effects on visuomotor consolidation were expected to be present after a night of sleep in all groups. Nap-dependent effects on visuomotor performance were not found immediately after napping and also not after an additional night of sleep in individuals after stroke.

Participants in the current study spend the majority of their nap-time in light, NREM sleep stages, including N1 and N2. Evidence on the role of NREM sleep in memory consolidation of motor adaptation tasks remains inconsistent (47). Particularly sleep spindles, a major characteristic of sleep stage N2, were previously correlated with sleep-dependent improvements in motor tasks in young adults (8, 48) and naps rich in N2 sleep facilitates delayed motor consolidation in older adults after an additional night of sleep (32). While most young adults show an increase in density of sleep spindles after motor learning, older adults do not (49) and also in individuals after stroke, no significant correlation could be found for offline learning and time spend in N2 (23). Thus, results from a young population may not be readily transferable to another, older population, especially when considering changes accompanying healthy aging. These age-related changes include architectural changes such as a decrease in sleep efficiency (50) or spindle occurrence, but also spectral power as seen in slow wave activity (51). NREM slow waves support memory transformation of the hippocampus to neocortical areas (51). Accelerated forgetting, as seen in the population of older adults after a night of sleep has previously been associated with age-related impairment of NREM sleep (52). In chronic stroke patients even poorer sleep efficiency, unrelated to lesion size and in comparison to age-matched healthy controls has been reported for nighttime sleep (53). A recent meta-regression analysis underlined the importance of sleep architecture and reported a positive correlation between sleep efficiency and the effect size for sleep-based memory consolidation of declarative and procedural tasks in healthy older adults (54). In the current experiment, sleep quality did not differ between both populations (PSQI: stroke: 3.3 ± 2.9 points, old: 3.9 ± 2.1 points).

### Population-related aspects

A significant main effect was found in both populations when comparing daytime and nighttime offline learning, with a general negative trend for performance scores during a night of sleep. This difference emerged as older adults significantly deteriorated after a night of sleep, whereas individuals after stroke were able to maintain their performance level throughout a day– and nighttime break. The initial offline improvement during daytime in contrast to subsequent offline deterioration (nighttime) was found in both populations. The finding may be linked to the stage of learning and its ability to benefit from sleep (55). Offline learning periods during initial stages of learning are more likely to elicit a larger change than during later stages when less improvement is expected (56). The significant difference in deterioration over a night of sleep may also be linked to the participants' age. The population of individuals after stroke (62.0 ± 7.7 years) was not as old as the individuals of the healthy older population (71.5 ± 5.6 years). Even though this factor was not significant when included into the model to calculate offline change and there was no correlation between age and any learning variable, it may be that memory-related brain areas of the older population underwent functional and possible structural changes disrupting the sleep-enhanced dialogue of hippocampal and neocortical regions (52). Processes of plasticity are not solely limited to the initial time after stroke but can continue in the chronic phase (57, 58). One might speculate that an injury to the brain such as an ischemic stroke can facilitate a change in functional plasticity, possibly leading to more effective or less sleep-affected processes of consolidation. Functional (possibly compensatory) differences, including a decreased BOLD signal of the dorsolateral prefrontal cortex in age- and sex-matched healthy control compared to individuals after stroke after learning of an implicit joystick-task were reported in a previous study (59). One might also argue that the difference arises from structural changes resulting from the rehabilitative training, similar to findings from a 3-month juggle training in adults showing increased gray matter density (60, 61). One study on sleep and motor learning in individuals after stroke, brain tumor or after a trauma, found that prefrontal lesion locations did not hamper offline sequence learning while parietal stroke locations did (22). A difference related to stroke location may thus be possible. The difference between both populations with older adults showing a decline of knowledge overnight and individuals after stroke not, is similar to a finding from previous studies in comparably aged stroke patients (21, 23). Latter did not only find a stagnation of performance but even more, a positive effect of sleep. One major difference to these prior studies was the hand used to perform the task. Previous studies in individuals after stroke focused on sequence learning and instructed individuals to make use of the individually preferred (22) or the ipsilesional hand (21, 23, 25). This was done to separate motor execution abilities from motor learning capabilities (62, 63). As motor execution of the ipsilesional hand is also affected by stroke (64–66), the change of performing hands may not be the optimal solution to this common obstacle in stroke research. In addition, it does not resemble daily rehabilitation practice and is therefore of limited use when transferring basic research back to bedside. The current study population consists of well-recovered individuals after stroke, similar to Siengsukon et al. (23) with respect to functional scores (F-M). Lower F-M scores (less-well recovered) are reported in prior publications (21, 24) of the same research group. While the latter and others recruited individuals at a late stage, around 6–7 years after stroke (21, 23, 24), the current study population performed the task around 2.4 years after a stroke, both time-points being classified as the chronic stage after stroke. The task specifications limited recruitment of individuals after stroke to a small extent, individuals with plegic arms could not be recruited (F-M range: 21–60 points). Some participants (*n* = 7) were unable to perform the 9-HPT, the lacking fine motor skills did not appear to hamper the task execution using a joystick.

### Task-related aspects

The present lacking sleep-dependent performance improvements may also be task-related. This study implemented a motor adaptation task which requires movement planning along with precise movement execution. The large rotation angle used make it very likely that explicit strategies were used during movement planning and execution. Even though it has been implemented as is in Backhaus et al. (35, 42) and similarly (27) in prior studies, the results should not be compared to the more common sequence learning paradigms directly. It was implemented as a more functional task, with respect to stroke rehabilitation. Thus, similar to an even more applied task (15), comparable studies are lacking. The introduced random blocks, as commonly done in sequence learning studies, did not provide additional value.

Next to the main outcome, number of collected targets, also the distance traveled during task execution was recorded. The distance traveled to each target, per trial may be more capable of representing the large movement variability in stroke patients. Less collected targets were usually the result of longer distances traveled while more collected targets implied less distance traveled. The previous analysis, using the number of collected targets within a certain time frame, was able to detect a difference between day– and nighttime offline learning [*F*_(1, 27)_ = 6.7, *p* = 0.015], this could not be shown using the distance traveled.

Sleep was found to selectively enhance memory with increased future relevance (67), or selectively prevent consolidation of tasks with conflicting future relevance, as has been described for an inverse bicycle steering task (20). As the relevance of this specific type of joystick control for daily life activities can be expected to be rather low in both populations, it was nevertheless an engaging task which once more, since the start of rehabilitation in individuals after stroke, required the adaptation of a motor program to external stimuli.

### Study limitations

The common approach to show the effect of sleep on memory consolidation of a previously learned motor task is to compare overnight sleep (learning p.m., retest a.m.) to an equivalent period of daytime wakefulness (learning a.m., retest p.m.). This type of study design is inevitably influenced by circadian factors, which may (18, 34, 56) or may not (13) alter sleep-dependent consolidation. We implemented a daytime nap design during which all participants learn the motor task during the same time of the day (session 1: wake: 12:49 p.m. ± 38 min, short nap: 12:52 p.m. ± 33 min, long nap: 1:22 p.m. ± 30 min, session 2: wake: 2:08 p.m. ± 38 min, short nap: 2:16 p.m. ± 31 min, long nap: 3:53 p.m. ± 30 min, session 3: wake: 10:29 a.m. ± 43 min, short nap: 10:15 a.m. ± 56 min, long nap: 10:25 a.m. ± 46 min).The first learning session was scheduled in average 6 h (wake: 5.58 h ± 58 min, short nap: 5.56 h ± 88.5 min, long nap: 7 h ± 76.8 min) after scheduled wake-up of the participants. As a weakness of the nap design, the homeostatic sleep drive may be altered in one trial arm (participants remaining awake vs. nappers), leading to changes of alertness, which may possibly impact performance (68). Also in the current study, the subjective level of sleepiness differed between the groups following the midday break. However, this subjective difference appeared not to have a significant impact on objective measures, such as motor performance scores or in visual reaction time scores, both showing no significant differences between the groups at any point in time. Nonetheless, the choice of study paradigm leads to a few limitations of this study, which should be addressed.

A limitation, linked to the study setup, is the difference in time until retest (session two) for the long nap group in comparison to the wake and short nap group. Participants in the long nap group restarted the experiment later than both other groups. This was due to the double amount of sleep time paired with the additional time the participants were given to reduce any possible effects emerging from sleep inertia. The latter was well controlled for, as seen in the non-significant differences between groups in reaction times, as well as, in subjective sleepiness. Previous studies on reaction times and subjective sleepiness implemented multiple retests after learning and could not show any effect of the elapsed time (69). This difference in elapsed time until retest did therefore, most likely, not alter the motor performance or the results of this study substantially.

A second limitation concerns the control condition “awake.” A more strict protocol could have been implemented by having the participants lie in bed, in a darkened room, without falling asleep. Blue light of the television and the upright position may have influenced processes of consolidation.

Third, as the effect of napping remained far below the expected effect an increase in sample size would have been beneficial to decrease chances for type II errors. However, large and clinically relevant effects are unlikely to be missed in the present setting.

Lastly, while participants were screened for sleep disorders they did not undergo a night with polysomnographic recordings to fully exclude possible non-detected disorders. Disordered sleep is common in stroke patients and might impact learning (70, 71).

## Conclusion

Individuals in the chronic stage after stroke are capable to show performance improvements of tasks executed with the contralesional (stroke impaired) limb. In line with results from younger and older adults in an identical setup (35, 42), midday naps, in comparison to wakefulness, did not enhance nor deteriorate this motor outcome directly after the nap or after an additional night of sleep. The performance did not differ significantly between the groups (wake, short nap, long nap) at any time point. While for one, it may be that, similar to both daytime nap conditions, wakeful rest allowed neural replay [as seen in (72)], a possible mechanism for memory consolidation (73), it may also be that the previously described positive effect of sleep on memory consolidation is the result of multiple processes (fatigue during training, averaging methods, reactive inhibition etc.) (56), other than sleep.

A comparison of performance in healthy older adults and individuals after stroke highlighted changes in offline consolidation during a night of sleep. Individuals after stroke managed to maintain their level of performance in contrast to the performance deterioration in older adults after a night of sleep.

One-time daytime sleep after motor training did not influence behavioral gains and renders it unlikely that a one-time “nap intervention” exerts a clinically relevant adjuvant effect on hand-focused visuomotor training in a neurorehabilitation setting with patients in the chronic phase after stroke.

## Author contributions

WB, FH, HB, and CG contributed conception and design of the study. WB finalized the experimental design, collected all data and performed the statistical analysis. WB wrote the first draft of the manuscript. All authors contributed to manuscript revision, read and approved the submitted version.

### Conflict of interest statement

The authors declare that the research was conducted in the absence of any commercial or financial relationships that could be construed as a potential conflict of interest.
