# Supplementary material for: Can Daytime Napping Assist the Process of Skills Acquisition After Stroke?
Source: Front Neurol. 2018 Nov 22;9:1002. doi: 10.3389/fneur.2018.01002 (PMC6262055; doi:10.3389/fneur.2018.01002)
Supplement: Supplementary file 1 [file Table_1.DOCX]

**Supplementary Material**

|  |  | wake | nap | long nap |
| --- | --- | --- | --- | --- |
| extended media infarction |  | 5 | 4 | 6 |
| corona radiata |  | 2 | 2 | 1 |
| internal capsula |  | 2 | 1 | 2 |
| Thalamocapsular |  | 3 | 1 | 0 |
| basal ganglia |  | 2 | 1 | 0 |
| motor cortex |  | 3 | 1 | 2 |
| postcentral gyrus |  | 0 | 0 | 1 |
| Insular |  | 1 | 2 | 1 |
| Pons |  | 0 | 2 | 0 |

***suppl. Table 1****: Lesion locations with affected brain regions per group. Multiple lesion locations were possible.*
